# Outcome of Second Primary Malignancies Developing in Multiple Myeloma Patients

**DOI:** 10.3390/cancers15174359

**Published:** 2023-09-01

**Authors:** Irit Avivi, David H. Vesole, Julio Davila-Valls, Lidia Usnarska-Zubkiewicz, Magdalena Olszewska-Szopa, Vibor Milunovic, Bartłomiej Baumert, Bogumiła Osękowska, Anna Kopińska, Massimo Gentile, Borja Puertas-Martinez, Paweł Robak, Edvan Crusoe, Luis Gerardo Rodriguez-Lobato, Małgorzata Gajewska, Gergely Varga, Michel Delforge, Yael Cohen, Alessandro Gozzetti, Camila Pena, Chaim Shustik, Gabor Mikala, Klara Zalac, H. Denis Alexander, Peter Barth, Katja Weisel, Joaquín Martínez-López, Anna Waszczuk-Gajda, Mateusz Krzystański, Artur Jurczyszyn

**Affiliations:** 1 Department of Hematology, Tel Aviv Sourasky Medical Center and Faculty of Medicine, Tel Aviv University, Tel Aviv 6997801, Israel; 2 Hackensack University Medical Center, New Jersey Medical School, Rutgers University, Hackensack, NJ 07601, USA; 3 Hospital Nuestra Señora de Sonsoles, 05004 Ávila, Spain; 4 Department of Hematology, Blood Neoplasms and Bone Marrow Transplantation, Wroclaw Medical University, 50-556 Wroclaw, Poland; 5 Division of Hematology, Clinical Hospital Merkur, 10000 Zagreb, Croatia; 6 Department of General Pathology, Pomeranian Medical University, 70-111 Szczecin, Poland; bbaumert@pum.edu.pl (B.B.);; 7 Department of Hematology and Bone Marrow Transplantation, Medical University of Silesia, 40-032 Katowice, Poland; 8 Hematology Unit AO of Cosenza, Cosenza and Department of Pharmacy, Health and Nutritional Sciences, University of Calabria, 87036 Rende, Italy; 9 Instituto de Investigación Biomédica de Salamanca (IBSAL), Cancer Research Center-IBMCC (USAL-CSIC), CIBERONC, University Hospital of Salamanca, 37007 Salamanca, Spain; 10 Department of Hematology, Copernicus Memorial Hospital, Medical University of Lodz, 90-752 Lodz, Poland; 11 Universidade Federal da Bahia, Hospital Universitário Professor Edgar Santos, Serviço de Hematologia, Salvador 40110-909, BA, Brazil; 12 Amyloidosis and Multiple Myeloma Unit, Department of Hematology, Hospital Clínic of Barcelona, IDIBAPS, 08036 Barcelona, Spain; 13 Department of Internal Medicine and Hematology, Military Institute of Medicine, 04-141 Warsaw, Poland; 14 Department of Internal Medicine and Haematology, Semmelweis University, 1085 Budapest, Hungary; 15 University Hospitals (UZ) Leuven, 3000 Leuven, Belgium; 16 Department of Medical Science, Surgery and Neuroscience, University of Siena, 53100 Siena, Italy; 17 Sección Hematología, Hospital del Salvador, Santiago 13123, Chile; 18 Royal Victoria Hospital, McGill University Health Centre, Montreal, QC H4A 3J1, Canada; 19 Department of Hematology and Stem Cell Transplantation, National Institute for Hematology and Infectious Diseases, South Pest Central Hospital, 1097 Budapest, Hungary; gmikala67@gmail.com; 20 Department of Hematology, Clinics for Internal Medicine, University Hospital Center “Sestre Milosrdnice”, 10000 Zagreb, Croatia; 21 Personalised Medicine Centre, School of Medicine, Ulster University, Derry/Londonderry BT47 6SB, UK; d.alexander@ulster.ac.uk; 22 Department of Medicine, Warren Alpert Medical School, Brown University, Providence, RI 02912, USA; 23 Department of Oncology, Hematology and Bone Marrow Transplantation with Section Pneumology, University Medical-Center Hamburg-Eppendorf, 20246 Hamburg, Germany; 24 Nieves Lopez-Muñoz Hospital, 28029 Madrid, Spain; jmarti01@ucm.es; 25 Department of Hematology, Oncology and Internal Diseases, Warsaw Medical University, 02-097 Warsaw, Poland; 26 Independent IT Specialist, 31-864 Krakow, Poland; 27 Department of Hematology, Jagiellonian University Medical College, 31-155 Crakow, Poland; arturjurczyszyn@gmail.com

**Keywords:** second primary malignancy, SPM, multiple myeloma, therapy

## Abstract

**Simple Summary:**

The emergence of new therapeutic agents for multiple myeloma (MM) over the last 2 decades has resulted in a significant improvement in overall survival (OS). However, this improvement might be associated with the increased incidence of second primary malignancies (SPMs). Most studies in the field reviewed patients that participated in phase 2–3 clinical studies, focusing on the incidence of SPMs. The current study evaluated the characteristics, management, and outcomes of MM patients diagnosed with SPMs outside clinical studies. In our study, we present real-world data of 165 MM patients that were diagnosed with SPM during the course of their disease; we offer detailed data on SPM characteristics and management, as well as valuable insights into the management of MM post-SPM detection and the actual prognosis of MM patients following SPM diagnosis.

**Abstract:**

Background: There is an increased risk of second primary malignancies (SMPs) in patients with multiple myeloma (MM). This multinational ‘real-world’ retrospective study analyzed the characteristics and outcomes of MM patients that developed SPMs. Results: 165 patients were analyzed: 62.4% males; 8.5% with a prior cancer; 113 with solid SPMs, mainly ≥stage 2; and 52 with hematological SPM (hemato-SPM), mainly MDS/AML. Patients with hemato-SPM were younger (*p* = 0.05) and more frequently had a prior AutoHCT (*p* = 0.012). The time to SPM was shorter in the older (>65 years) and more heavily pretreated patients. One hundred patients were actively treated at the time of SPM detection. Treatment was discontinued in 52, substituted with another anti-MM therapy in 15, and continued in 33 patients. Treatment discontinuation was predominant in the patients diagnosed with hemato-SPM (76%). The median OS following SPM detection was 8.5 months, and the main cause of death was SPM. A poor ECOG status predicted a shorter OS (PS 3 vs. 0, HR = 5.74, 2.32–14.21, *p* < 0.001), whereas a normal hemoglobin level (HR = 0.43, 0.19–0.95, *p* = 0.037) predicted longer OS. Conclusions: With the continuing improvement in OS, a higher proportion of MM patients might develop SPM. The OS following SPM diagnosis is poor; hence, frequent surveillance and early detection are imperative to improve outcomes.

## 1. Introduction

The emergence of new therapeutic agents for multiple myeloma (MM) over the last 2 decades has resulted in a significant improvement in overall survival (OS). However, there has also been a simultaneous increase in the incidence of subsequent second primary malignancies (SPMs) [1,2,3], which is attributed to the immunosuppressive milieu characterizing MM, together with the exposure to anti-neoplastic agents, particularly high-dose melphalan in patients undergoing an autologous hematopoietic stem cell transplantation (AutoHCT) [3,4,5]. However, most of the data regarding the characteristics and outcomes of the patients that developed SPMs derive from clinical studies that excluded patients with prior malignancies, either recent or active. Moreover, most of these studies have focused on the incidence of SPMs, instead of analyzing the characteristics, management, and outcomes of these patients. Lastly, the patients enrolled in clinical studies were required to discontinue their current anti-MM therapy at the time of the diagnosis of SPM. Subsequently, data regarding MM management and the outcomes of these patients after SPM detection are lacking.

The current study evaluated the characteristics, management, and outcomes of MM patients diagnosed with SPMs outside clinical studies.

## 2. Materials and Methods

Myeloma databases of all 25 participating centers were retrospectively screened for consecutive patients that were diagnosed with SPM between January 1996 and January 2021 and were treated with at least one line of anti-MM treatment regime prior to the detection of SPM. SPM was defined as a new malignancy that was detected at least 6 months after the initiation of first line anti-MM therapy. Patients who experienced recurrence or progression of cancer that was documented prior to MM diagnosis were excluded from the analysis. Patients diagnosed with a non-melanoma skin cancer were excluded. The institutional review boards of all the participating centers approved the study. The data were collected from the patients’ medical records and included: demographics, concomitant comorbidities, myeloma-related parameters at diagnosis, treatment regimens that were administered prior to SPM detection (including prior autologous hematopoietic cell transplantation (AutoHCT), time to SPM diagnosis (solid or hematological), SPM histology and staging (determined by the recommended staging system for each type of malignancy), SPM management, MM management in response to SPM detection, survival, and causes of death. The factors associated with shorter time to SPM and shorter survival following SPM diagnosis were analyzed.

### Statistics

Statistical analysis was performed in the RStudio (3 February 2022, build 492) together with survival analysis and data processing/visualization packages. To compare characteristics between our subgroups we used Pearson’s chi-squared test and the Wilcoxon rank sum test. The Cox proportional hazard regression method was performed for univariate analysis. Factors with *p*-values < 0.05 in the univariate models were included in the multivariate Cox regression model. All estimates have 95% confidence intervals and two-sided *p*-values. Kaplan–Meier charts were used to visualize the results.

## 3. Results

### 3.1. Patient Characteristics

Two hundred and one consecutive MM patients, all diagnosed with a new SPM, detected at least 6 months after the initiation of first line anti-MM therapy, were extracted from the MM databases of the participating centers. Thirty-six patients were excluded from the analysis as they had non-melanoma skin cancers. One hundred and sixty-five MM patients, 64.2% males (n = 103), including 8.5% with a prior history of a different cancer, were included in the analysis (the patient characteristics are shown in Table 1). The median ages at MM and at SPM diagnosis were 65 years (57–70) and 70 years (64–75), respectively. Thirty-percent presented with International Staging System 3. Seventy-eight percent (n = 128) had previously been exposed to proteasome inhibitors (PIs); 86.7% (n = 143) to immunomodulating (IMiDs) agents (including 73.3%, n = 121, exposed to both); and 8% (n = 13) to monoclonal antibodies (MOABs). Half of the patients (n = 83) underwent an AutoHCT and 39% (n = 64) received maintenance therapy (54 with IMiDs and 10 with PIs). The median number of treatment lines prior to SPM detection was two (range 1–7). One hundred and thirteen patients were diagnosed with solid SPMs and fifty-two with hemato-SPM.

There were no statistically significant differences in terms of gender, incidence of prior cancer, exposure to specific anti-MM agents, or the employment of maintenance therapy between the patients that developed solid SPM and those that developed hemato-SPMs (Table 1). However, the patients diagnosed with solid SPM were older at MM diagnosis (*p* = 0.05) and tended to have concomitant diabetes (22% vs. 9.6%, *p* = 0.08), whereas patients that developed hemato-SPM were more likely to have undergone a prior AutoHCT (65.4% vs. 43.4%, *p* = 0.012).

### 3.2. SPM Characteristics

One hundred and thirteen patients had solid SPM: colorectal (n = 17; 15%), lung (n = 16; 14.2%), and prostate (n = 10; 9%), followed by breast, melanoma, bladder, and pancreatic cancer—each one occurring in nine (8%) patients. (Table S1A, Supplementary File, presents the SPM subtypes). Disease stage was ≥II in two-thirds of the patients that were diagnosed with solid SPMs, for whom a disease stage was available, (61/92), including 15% (n = 14) that were diagnosed with stage II, 24% (n = 22), that were diagnosed with stage III, and 27% (n = 25) that were diagnosed with stage IV disease.

Fifty-two patients (31.5%) developed hemato-SPMs, including thirty-six patients that developed acute myeloid leukemia (AML)/myelodysplastic syndrome (MDS) or MDS/myeloproliferative disease (MPN), five with acute lymphoblastic leukemia, and eleven that developed non-Hodgkin lymphoma (NHL) (Table S1A). Of note, the patients with leukemia and MDS commonly exhibited high-risk genetic signatures [6], (20/21 of the leukemia/MDS patients for whom karyotype and molecular analysis were available had poor risk features (Table S1B, Supplementary File)), whereas those diagnosed with NHL mainly presented with Ann Arbor stage 4 disease (73%; 8/11).

The median time from MM diagnosis to solid SPM was 57 months, and to hemato-SPM, it was 63 months (*p* = 0.3). Age ≥ 65 years (HR = 2.53, CI95% 1.83–3.51, *p* < 0.001); a history of prior cancer (HR = 2.36, CI95% 1.35–4.1, *p* = 0.003); a higher beta-2 microglobulin (B_2_M) at diagnosis (HR = 1.48, CI95% 1.01–2.18, *p* = 0.04); and concomitant type 2 diabetes mellitus (T2DM) (HR = 1.82, CI95% 1.21–2.72, *p* = 0.004) were all associated with a shorter time to SPM detection. In contrast, good performance status (0 vs. higher) (HR = 0.62, CI95% 0.42–0.93, *p* = 0.019); fewer prior lines of therapy (≤2 vs. ≥3) (HR = 0.41, CI95% 0.29–0.57, *p* < 0.001); hemoglobin level ≥ 12 g/dL (HR = 0.69, CI95% 0.49–9.99, *p* = 0.042); platelet count ≥ 150,000/microliter (HR = 0.52, CI95% 0.3–0.83, *p* = 0.006); and lack of concomitant comorbidities (HR = 0.56, CI95% 0.4–0.8, *p* = 0.001) were all associated with a longer time to SPM in univariate analysis (Table 2A).

**Figure 1:**
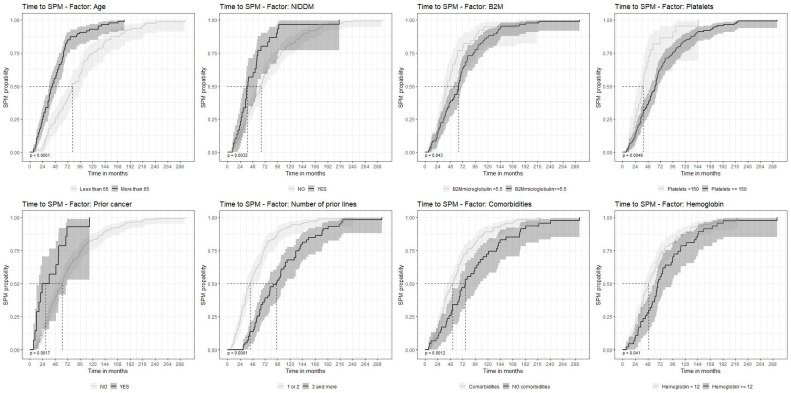
Time to second primary malignancy depending on the factors selected (univariate analysis). Factors in order: age; NIDMM (non-insulin-dependent diabetes); B_2_M (beta-2 microglobulin); platelet count; prior history of cancer; prior myeloma therapy; comorbidities; hemoglobin levels. SPM—second primary malignancy.

Multivariate analysis confirmed that age ≥ 65 years (HR = 2.87, CI95% 0.25–0.59, *p* < 0.001) was associated with a shorter time to SPM. Fewer lines of prior therapy (HR = 0.38, CI95% 0.25–0.59, *p* < 0.001), normal platelet counts (HR = 0.58, CI95% 0.35–0.94, *p* = 0.026), and hemoglobin levels (HR = 0.63, CI95% 0.4–1.00, *p* = 0.048) were associated with a longer time to SPM detection (Table 2B, Figure 1).

The comparison of the characteristics between time to solid SPM and time to hemato-SPM found that age, a history of prior cancer, and the number of prior lines of therapy had a significant impact on both cancer subtypes (Table S2A,B, Supplementary Material). Concomitant T2DM (*p* = 0.029) and increased B_2_M level at diagnosis (*p* = 0.012) were associated with a shorter time to the detection of solid SPM, whereas a lower PLT count at an MM diagnosis (<150,000) (*p* = 0.021) and a higher number of comorbidities (*p* = 0.003) predicted a shorter time to hemato-SPMs.

### 3.3. SPM and MM Management following SPM Detection

Seventy-six percent (n = 126) received a specific treatment for their SPM, including seventy-eight percent (n = 88) of those that were diagnosed with solid SPMs and seventy-three percent (n = 38) of those that developed a hemato-SPM (details on anti-SPM therapy are provided in the Supplementary File, Table S3).

One hundred patients were receiving an anti-MM therapy at the time of SPM diagnosis (seventy-five diagnosed with solid SPM and twenty-five diagnosed with hemato-SPM) (Table 1). Treatment remained unchanged in 33% (n = 33), changed to a new anti-MM therapy in 15% (n = 15), and was discontinued in 52% (n = 52) patients. Discontinuation of any anti-MM therapy was more commonly reported in patients with hemato-SPM (76% vs. 44% in patients with solid-SPMs). Only 4% of patients with hemato-SPM continued their current anti-MM therapy vs. 43% of those diagnosed with solid SPM (Table 3). Detailed data on MM management in response to SPM detection are provided in Table S4A, Supplementary File.

### 3.4. Survival

Within a median follow-up period of 157 months following MM diagnosis and 31 months following SPM detection, 106 died. Sixty patients (56.6%) died of SPM; seventeen died of MM progression (including three that experienced simultaneous progression of their SPM); sixteen died due to other causes, unrelated to MM or SPM progression; and in thirteen cases the cause of death remained unknown. SPM was the most common cause of death in both the patients with solid SPMs and those with hemato-SPMs, being responsible for 43/74 deaths in the solid SPM cohort, and 17/32 deaths in the hemato-SPM cohort. However, MM-related deaths were more commonly documented in the patients in the solid SPM group, with 14/74 deaths, compared with 3/32 in the hemato-SPM cohort (including the cases with simultaneous progression of solid SPM and hemato-SPM).

**Figure 2:**
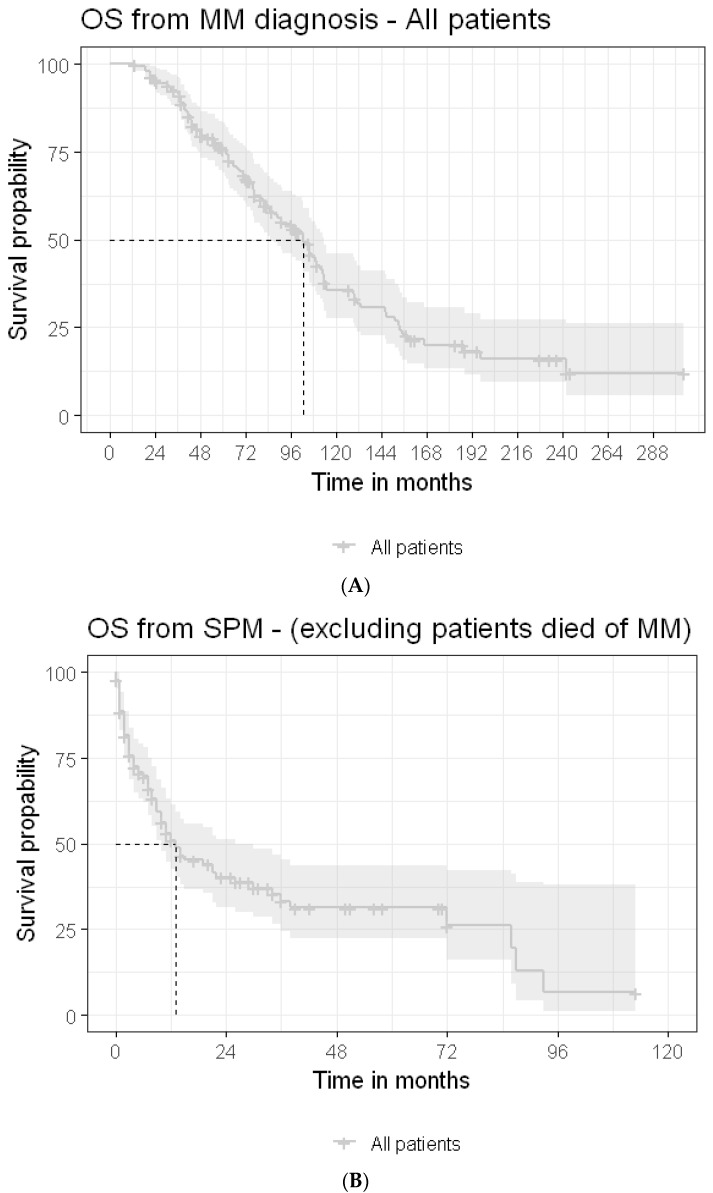
(**A**) Median overall survival following MM diagnosis in all SPM patients. OS—overall survival; MM—multiple myeloma. (**B**) Median overall survival following second primary malignancy, excluding patients that died of myeloma. OS—overall survival; MM—multiple myeloma; SPM—second primary malignancy.

The median OS periods following MM diagnosis (Figure 2A) and following SPM detection for the entire cohort were 77 months and 8.5 (3–22) months, respectively, with no statistically significant difference between patients with solid SPMs and those with hemato-SPMs: 10 (range: 3–26) months vs. 7 (range: 2–14) months. Multivariate analysis determined that the HB level ≥ 12 gr/dL was associated with longer OS (HR = 0.38, CI95%, 0.18–0.81, *p* = 0.013), whereas poor PS predicted shorter OS (for PS = 3, HR = 6.38, CI95%, 2.73–14.9, *p* < 0.001) (Table 4) (univariate analysis is presented in Table S4B,C, Supplementary File).

**Figure 3:**
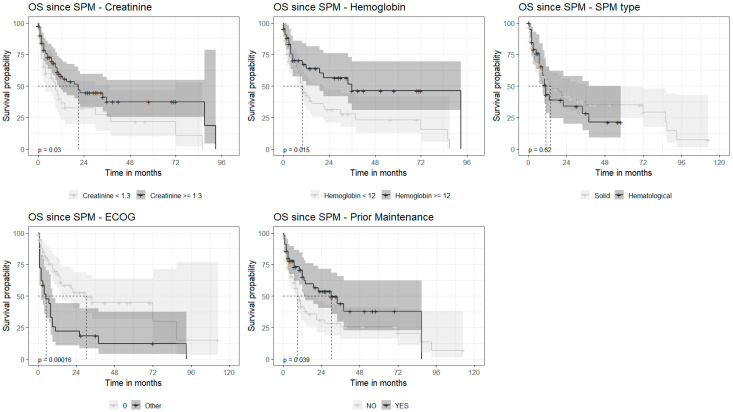
Overall survival depending on the factors selected (univariate analysis). Factors in order: creatinine; hemoglobin level; SPM (second primary malignancy) type; ECOG—Eastern Cooperative Oncology Group grade; prior maintenance. OS—overall survival.

Repeated analysis, excluding the patients that died of MM, revealed very similar results, with a median OS of 8 (range) months (Figure 2B). Univariate analysis found that increased creatinine level (≥1.3 mg/dL) (HR = 1.73, CI95%, 1.05–2.85, *p* = 0.032) and poorer performance status were associated with shorter survival: 0 vs. ≥1 (HR = 7.88, CI95%, 2.8–22, *p* < 0.001). In contrast, a normal hemoglobin level at diagnosis (HR = 0.51, CI95%, 0.30–0.89, *p* = 0.017) and the administration of any maintenance therapy post-autograft (HR = 0.54, CI95%, 0.32–0.92, *p* = 0.023) predicted a longer OS. There were no statistically significant differences in OS between the patients diagnosed with solid SPMs and those with hemato-SPMs (*p* = 0.52) (Figure 3). Multivariate analysis revealed poor PS (PS 3 vs. 0, HR = 5.74, 2.32–14.21, *p* < 0.001) to be associated with shorter OS following SPM detection, whereas the HB level ≥ 12 g/dL at presentation (HR = 0.43, 0.19–0.95, *p* = 0.037) and any administration of maintenance therapy (HR = 0.51, CI95%, 0.27–0.94, *p* = 0.031) were associated with longer OS (Table 4).

## 4. Discussion

Myeloma patients have increasing OS due to the availability of new therapeutic agents over the past 15 years [7]. Given this improved survival, there might be an increased risk of the development of SPMs. Our study was to evaluate the ‘real-world’ characteristics, management, and outcomes of MM patients with SPMs outside of the clinical trial setting. Our study was not designed to define the incidence of SPM in MM patients, nor to define risk factors for SPM development.

According to our findings, 8% of the patients had a former malignancy prior to MM development; these results are in line with some of the previous studies, which suggested that having a previous SPM might be a risk factor for SPM development [8,9,10]. However, a recently published large population-based study failed to demonstrate a higher risk for SPMs in patients with a former primary malignancy [11]. The patients diagnosed with solid SPMs were generally older and had a higher frequency of concomitant T2DM compared to hemato-SPM, reflecting the increasing incidence of metabolic diseases with aging and/or the contribution of T2DM and its associated pro-inflammatory milieu to the development and progression of solid SPMs [12,13]. As expected, patients that developed hemato-SPM were more commonly exposed to high-dose therapy and subsequent lenalidomide maintenance, supporting prior reports that suggested that melphalan exposure increases the risk for AML/MDS [14,15]. The mechanism of this increased risk has been postulated to be caused by the promotion of the progression of a preexisting clonal hematopoiesis indirectly [16] or directly [17] (occurring in up to 21.6% of newly diagnosed MM patients) [18] into clinically significant myelodysplasia or leukemia. The most common solid SPMs were colon, lung, and prostate cancers, which were well matched with their frequency order in the general population [19]. The majority of patients diagnosed with solid SPMs presented with an advanced disease stage, possibly reflecting the immunosuppressive environment accompanying MM [20] and the tumorigenic/immunosuppressive effects of anti-MM agents, especially alkylating agents [21], which potentially promote the progression of SPMs.

Interestingly, in contrast to their frequencies in the general population [22], but in line with prior studies that evaluated SPMs in patients with MM [23], the most common hemato-SPMs were AML and MDS rather than NHL [24,25], caused by the progression of occult BM clonal hematopoiesis(ref) and/or treatment-related toxicity, mainly AutoHCT [17]. 

The time to SPM diagnosis was significantly shorter in older patients, reflecting the higher incidence of most cancers with aging and/or the progression of an occult cancer/pre-cancerous condition promoted by the immunosuppressive milieu accompanying both aging [26] and MM. A history of a prior cancer was associated with shorter time to SPM, an observation which is in line with the higher risk for additional malignancies in patients with a prior history of cancer [8]. Interestingly, concomitant comorbidities, especially preexisting T2DM, also tended to be associated with shorter time to SPM diagnosis, (particularly with solid SPM), supporting previous studies that found a linkage between T2DM and cancer development [27]. Additionally, a low PLT count was found to be associated with a shorter time to hemato-SPMs, suggesting a pre-existing MDS. 

Diagnosis of SPM led to cessation of any anti-MM therapy in half of the patients that were actively treated at the time of SPM diagnosis. Considering the huge diversity in SPM subtypes, the variability in SPM management, the impact of MM status at SPM detection on clinical decisions, and the insufficient data regarding the potential contribution of ongoing vs. discontinuing therapy to SPM evolution, it is almost impossible to generate recommendations regarding MM management in response to the detection of SPM. Almost none of the patients with hemato-SPM continued their ongoing anti-MM therapy and most discontinued anti-MM altogether. This “treatment withdrawal” strategy may be due to the initiation therapy directed against the SPM and/or the physicians “fear” of continuing anti-MM therapy, particularly IMiDs; it was proposed that latter were associated with SPMs, particularly AML/MDS [28,29]. The potential impact of the chosen anti-MM management on OS remained unclear given that the most common cause of death in our cohort was SPM rather than MM progression and that the number of patients in each management cohort (e.g., cessation vs. substitution vs. continuation) was too small to enable this analysis. Moreover, considering that most of the data regarding SPMs arise from interventional prospective studies, requiring the cessation of the study drug in the case of SPM detection, the data on the optimal management of MM are generally lacking.

As previously reported by others, the OS following the detection of hemato-SPM was remarkably short [1,23,24]. This dismal outcome was true for the entire cohort and for a selected group of patients, excluding those who had already died due to MM progression.

However, in contrast to some previous ‘real world’ studies [1,30], the survival of our solid SPM patients was also very poor. These differences may be explained by the exclusion of patients with non-melanoma skin cancer from our series, differences in the study periods, the clinical stage of SPM, and the availability of treatment options. As expected, poor PS was associated with shorter OS following SPM detection. The administration of maintenance therapy following diagnosis was also associated with longer OS following SPM detection, potentially reflecting a “fitter population” of patients that were selected for AutoHCT and received maintenance post-autograft. A recently published study suggested that the development of SPM is also associated with short PFS [24] and probably reflects a consecutive weakening in the anti-MM therapy being employed, due to either its cessation, substitution with less effective treatment, or the administration of reduced treatment doses due to concurrent anti-SPM therapy [24].

Unfortunately, due to the short survival observed in our cohort after SPM detection and the lack of a matched control group of MM patients that received the same therapies but did not develop SPMs, it was impossible to assess the specific impact of SPM on PFS. Nevertheless, considering the frequent cessation of anti-MM therapy in response to SPM detection, it highly likely that these patients will also experience a shorter PFS compared to that of their MM counterparts that did not develop SPMs. Moreover, prior studies suggested that SPMs are higher in patients with cytogenetic abnormalities, which are, irrespectively, liable to cause shorter PFS and OS [24,31].

## 5. Conclusions

There is an absolute need to employ structured surveillance protocols for patients with MM that would enable an early detection of SPMs, particularly in patients presenting with the risk factors of advanced age and in more heavily treated patients. Our results are similar to those reported by the Netherlands Cancer Registry (diagnosed 1994 and 2013), which reported that older age and a history of prior SPMs were associated with shorter survival following SPM diagnosis [1].

Our study has several limitations. It is a retrospective study without the characteristics of bone marrow (especially on concomitant dysplastic changes, cytogenetics, or FISH data) or the exact duration of maintenance therapy with IMiDs; these are factors that might be associated with a risk for the development of a clinically significant MDS/AML. Nevertheless, our study provides a unique opportunity to review the characteristics and outcomes of MM patients diagnosed with SPM outside of clinical trials given that these patients often present with disseminated SPMs and experience a very poor outcome, one that is worse than generally reported in prospective clinical trials. With the continuing improvement in OS, the need for active surveillance (e.g., routine checkups in the skin clinic, mammography and ultrasound, and gynecological monitoring for women, PSA for males, colonoscopies and chest scans depending on individual risk factors, etc.) and the promotion of early detection and improved management of SPMs is highly necessary.

## Figures and Tables

**Table 1:** Patient characteristics.

		All SPMs n = 165	Solid SPMs n = 113	Hemato-l SPMs n = 52	*p*-Value
Demographics and prior medical history					
Age at MM diagnosis in years, median (range)		65 (57–70)	66 (57–71)	62.5 (56–68)	0.053
Age at SPM in years, median (range)		70 (64–75)	71 (64–77)	68 (64–72)	0.090
Sex	Female	62 (37.6%)	47 (41.6%)	15 (28.8%)	0.124
	Male	103 (62.4%)	66 (58.4%)	37 (71.2%)	
Prior cancer	NO	151 (91.5%)	103 (91.2%)	48 (92.3%)	1.000
	YES	14 (8.5%)	10 (8.8%)	4 (7.7%)	
T2DM	NO	135 (81.8%)	88 (77.9%)	47 (90.4%)	0.081
	YES	30 (18.2%)	25 (22.1%)	5 (9.6%)	
MM-related parameters					
Albumin (g/dL)	<3.50	51 (30.9%)	39 (34.5%)	12 (23.1%)	0.241
	≥3.5	79 (47.9%)	52 (46.0%)	27 (51.9%)	
	Missing	35 (21.2%)	22 (19.5%)	13 (25.0%)	
B_2_M level (mg/L)	<5.5	85 (51.5%)	61 (54.0%)	24 (46.2%)	1.0
	≥5.5	39 (23.6%)	28 (24.8%)	11 (21.2%)	
	Missing	41 (24.8%)	24 (21.2%)	17 (32.7%)	
ISS	1	40 (24.2%)	26 (23.0%)	14 (26.9%)	0.546
	2	42 (25.5%)	32 (28.3%)	10 (19.2%)	
	3	49 (29.7%)	35 (31.0%)	14 (26.9%)	
	Missing	34 (20.6%)	20 (17.7%)	14 (26.9%)	
Creatinine (mg/dL)	<1.3	99 (60.0%)	69 (61.1%)	30 (57.7%)	0.542
	≥1.3	41 (24.8%)	31 (27.4%)	10 (19.2%)	
	Missing	25 (15.2%)	13 (11.5%)	12 (23.1%)	
Hemoglobin (gr/dL)	<12	95 (57.6%)	67 (59.3%)	28 (53.8%)	0.695
	≥12	47 (28.5%)	35 (31.0%)	12 (23.1%)	
	Missing	23 (13.9%)	11 (9.7%)	12 (23.1%)	
Platelets (10^9^/L)	<150	22 (13.3%)	15 (13.3%)	7 (13.5%)	0.798
	≥150	114 (69.1%)	82 (72.6%)	32 (61.5%)	
	Missing	29 (17.6%)	16 (14.2%)	13 (25.0%)	
LDH level	Normal	90 (54.5%)	64 (56.6%)	26 (50.0%)	0.670
	Increased	36 (21.8%)	24 (21.2%)	12 (23.1%)	
	Missing	39 (23.6%)	25 (22.1%)	14 (26.9%)	
Time from first therapy to SPM (months)		60 (35–95)	57 (30–94)	63 (35.5–100.5)	0.312
Prior IMiD	NO	22 (13.3%)	15 (13.3%)	7 (13.5%)	1.000
	YES	143 (86.7%)	98 (86.7%)	45 (86.5%)	
Prior PI	NO	37 (22.4%)	29 (25.7%)	8 (15.4%)	0.163
	YES	128 (77.6%)	84 (74.3%)	44 (84.6%)	
Prior chemotherapy (excluding AutoHCT)	NO	47 (28.5%)	35 (31.0%)	12 (23.1%)	0.355
	YES	118 (71.5%)	78 (69.0%)	40 (76.9%)	
No. of prior therapies median, (range)		2 (1–7)	2 (1–7)	2 (1–7)	0.624
No. of prior therapies ≤ 2		106 (64.2%)	74 (65%)	32 (62%)	0.727
Prior AutoHCT	NO	82 (49.7%)	64 (56.6%)	18 (34.6%)	0.012
	YES	83 (50.3%)	49 (43.4%)	34 (65.4%)	
Prior maintenance *	NO	101 (61.2%)	72 (63.7%)	29 (55.8%)	0.391
	YES	64 (38.8%)	41 (36.3%)	23 (44.2%)	
MM treatment at the time of SPM detection	CHEMO	4 (2.4%)	2 (1.8%)	2 (3.8%)	0.639
	IMiD	55 (33.3%)	40 (35.4%)	15 (28.8%)	
	IMiD-CHEMO	5 (3.0%)	5 (4.4%)	0 (0.0%)	
	MOAB	8 (4.8%)	6 (5.3%)	2 (3.8%)	
	Not specified	15 (0.9%)	12 (11%)	3 (0.6%)	
	OTHER	1 (0.6%)	1 (0.9%)	0 (0.0%)	
	PI	7 (4.2%)	5 (4.4%)	2 (3.8%)	
	PI-IMiD	5 (3.0%)	4 (3.5%)	1 (1.9%)	
	No anti-MM therapy	65 (57.5%)	38 (34%)	27 (52%)	

SPM—second primary malignancy; AutoHCT—Autologous hematopoietic transplantation; B_2_M—B_2_ microglobulin; CHEMO—chemotherapy; IMiD—immunomodulating agents; ISS—International Staging System; LDH—lactate dehydrogenase; MM—multiple myeloma; No.—number; PI—proteasome inhibitor; T2DM—type 2 diabetes mellitus. * Fifty-four received maintenance with IMiD (n = 54) and 10 received PI. Hemoglobin, platelets, creatinine, albumin, and B_2_M levels were measured prior to initiation of any anti-MM treatment.

**Table 2:** (**A**) Univariate analysis—Factors predicting time to SPM detection following MM diagnosis. (**B**) Multivariate analysis—Factors predicting time to SPM following MM diagnosis.

(A)			
	HR	CI95%	*p*
Age ≥ 65 (years)	2.53	1.83–3.51	<0.001
Sex (Females)			
Males	1.11	0.81–1.52	0.52
Prior cancer	2.36	1.35–4.11	0.003
Concomitant T2DM	1.82	1.21–2.72	0.004
No concomitant comorbidities			
0	0.56	0.40–0.80	0.001
1	1.67	1.11–2.50	0.013
2	1.91	1.23–2.95	0.004
3	1.85	1.13–3.03	0.014
Creatinine level			
≥1.3 (mg/dL)	1.07	0.74–1.54	0.73
Albumin level			
≥3.5 (g/dL)	0.85	0.60–1.21	0.37
B2-microglobulin			
≥5.5 (mg/L)	1.48	1.01–2.18	0.044
ISS (1)			
2	1.24	0.80–1.92	0.34
3	1.42	0.93–2.17	0.10
Platelets > 150 (10^9^/L)	0.52	0.3–0.83	0.006
Hemoglobin ≥ 12 (gr/dL)	0.69	0.49–0.99	0.042
Any prior chemotherapy for MM	0.75	0.53–1.06	0.10
Any prior IMiD	0.93	0.59–1.46	0.74
Any prior PI	1.07	0.73–1.56	0.72
Any maintenance therapy	1.11	0.81–1.53	0.51
Prior AutoHCT	0.75	0.55–1.03	0.076
Number of prior lines (≤2 vs. ≥3)	0.41	0.29–0.57	<0.001
SPM type			
Solid vs. Hematolo	0.83	0.60–1.16	0.28
ECOG PS 0 vs. ≥1	0.62	0.42–0.93	0.019
(**B**)			
	**HR**	**CI95%**	***p***
Age < 65 vs. ≥65 (years)	2.87	0.25–0.59	<0.001
Prior cancer history	0.97	0.47–1.99	0.927
Number of prior lines <2 vs. ≥3	0.38	0.25–0.59	<0.001
PLT < 150 vs. >150 (10^9^/L)	0.58	0.35–0.94	0.026
B_2_-microglobulin ≥ 5.5 (mg/L)	1.14	0.73–1.78	0.575
Concomitant T2DM	1.56	0.98–2.49	0.059
Hemoglobin < 12 vs. ≥12 (gr/dL)	0.63	0.40–1.00	0.048

Hemoglobin, platelets, creatinine, albumin, and B_2_M levels were measured prior to initiation of any anti-MM treatment. ECOG PS was determined at SPM diagnosis. AutoHCT—Autologous hematopoietic cell transplantation; PS—ECOG performance status (PS); HT—hypertension; IMiD—immunomodulating agents; ISS—International Staging System; MM—myeloma; PI—proteasome inhibitor; SPM—second primary malignancy; T2DM—type 2 diabetes mellitus. PLT—platelets; NIDDM—non-insulin-dependent diabetes mellitus.

**Table 3:** Patient characteristics dependent on MM management in response to SPM detection. Characteristics of MM management depending on the type of SPM.

Characteristic		2—Change of Anti-MM Tx, n = 15 (%)	1—Discontinuation of Tx, n = 52 (%)	3—Continuation of Tx, N = 33 (%)	*p*-Value
SPM Type, n (%)	Solid (75)	10 (13.3)	33 (44)	32 (43)	<0.001
	Hemato (25)	5 (20)	19 (76)	1 (4)	

MM—multiple myeloma; SPM—second primary malignancy; Tx—therapy.

**Table 4:** Multivariate analysis—Factors predicting OS following SPM diagnosis; all patients.

	HR	CI95%	*p*
Any maintenance therapy	0.65	0.26–1.61	0.351
Creatinine level < 1.3			
≥1.3	0.74	0.40–136	0.334
ECOG PS 0			
1	2.62	1.16–5.91	0.020
2	5.16	1.76–15.08	0.003
3	6.38	2.73–14.90	<0.001
Hemoglobin < 12			
≥12	0.38	0.18–0.81	0.013

## Data Availability

Data available on request due to restrictions of privacy. The data presented in this study are available on request from the corresponding author. The data are not publicly available due to prrivacy.

## Supplementary Materials

The following supporting information can be downloaded at: https://www.mdpi.com/article/10.3390/cancers15174359/s1, Table S1 (A): SPM subtypes, Table S1 (B): Cytogenetic and molecular changes detected in 23 patients diagnosed with AML, ALL, MDS and MPN, Table S2 (A): Univariate analysis—Factors predicting time to solid SPM following MM diagnosis, Table S2 (B): Univariate analysis—Factors predicting shorter time to Hemato-SPM only, Table S3: Management of SPMs, Table S4 (A): Detailed characteristics of MM management in response to SPM detection, Table S4 (B): Univariate analysis—Factors predicting OS following SPM diagnosis for the entire cohort, Table S4 (C): Univariate analysis—Factors predicting OS following SPM diagnosis.

## References

[1] Brink M., Minnema M.C., Visser O., Levin M.D., Posthuma E.F.M.W., Broijl A., Sonneveld P., van der Klift M., Roeloffzen W.W.H., Westerman M. (2022). Increased mortality risk in multiple-myeloma patients with subsequent malignancies: A population-based study in the Netherlands. Blood Cancer J..

[2] Maclachlan K., Diamond B., Maura F., Hillengas J., Turesson I., Landgren C.O., Kazandjian D. (2020). Second malignancies in multiple myeloma; emerging patterns and future directions. Best Pract. Res. Clin. Haematol..

[3] Krishnan A.Y., Mei M., Sun C.L., Thomas S.H., Teh J.B., Kang T., Htut M., Somlo G., Sahebi F., Forman S.J. (2013). Second primary malignancies after autologous hematopoietic cell transplantation for multiple myeloma. Biol. Blood Marrow Transpl..

[4] Musto P., Anderson K.C., Attal M., Richardson P.G., Badros A., Hou J., Comenzo R., Du J., Durie B.G.M., Miguel J.S. (2017). Second primary malignancies in multiple myeloma: An overview and IMWG consensus. Ann. Oncol..

[5] Rosenberg A.S., Brunson A., Tuscano J., Jonas B.A., Hoeg R., Wun T., Keegan T.H.M. (2021). Effect of autologous hematopoietic stem cell transplant on the development of second primary malignancies in multiple myeloma patients. Blood Cancer J..

[6] Döhner H., Wei A.H., Appelbaum F.R., Craddock C., DiNardo C.D., Dombret H., Ebert B.L., Fenaux P., Godley L.A., Haserjian R.P. (2022). Diagnosis and management of AML in adults: 2022 recommendations from an international expert panel on behalf of the ELN. Blood.

[7] Gulla A., Anderson K.C. (2020). Multiple myeloma: The (r)evolution of current therapy and a glance into the future. Haematologica.

[8] Finkelstein D.M., Horick N.K., Ramchandani R., Boyd K.L., Rana H.Q., Bychkovsky B.L. (2019). Are rare cancer survivors at elevated risk of subsequent new cancers?. BMC Cancer.

[9] Zheng X., Li X., Wang M., Shen J., Sisti G., He Z., Huang J., Li Y.M., Wu A. (2020). Multidisciplinary Oncology Research Collaborative Group (MORCG). Second primary malignancies among cancer patients. Ann. Transl. Med..

[10] Liu Y., Hou H.A., Qiu H., Tang C.H. (2020). Is the risk of second primary malignancy increased in multiple myeloma in the novel therapy era? A population-based, retrospective cohort study in Taiwan. Sci. Rep..

[11] Jonsdottir G., Lund S.H., Bjorkholm M., Turesson I., Wahlin A., Mailankody S., Blimark C., Hulcrantz M., Porwit A., Landgren O. (2016). Survival in multiple myeloma patients who develop second malignancies: A population-based cohort study. Haematologica.

[12] Greten F.R., Grivennikov S.I. (2019). Inflammation and Cancer: Triggers, Mechanisms, and Consequences. Immunity.

[13] Tomic D., Shaw J.E., Magliano D.J. (2022). The burden and risks of emerging complications of diabetes mellitus. Nat. Rev. Endocrinol..

[14] Kazandjian D., Mo C.C., Landgren O., Richardson P.G. (2020). The role of high-dose melphalan with autologous stem-cell transplant in multiple myeloma: Is it time for a paradigm shift?. Br. J. Haematol..

[15] Jonsdottir G., Björkholm M., Turesson I., Hultcrantz M., Diamond B., Porwit A., Landgren O., Kristinsson S.Y. (2021). Cumulative exposure to melphalan chemotherapy and subsequent risk of developing acute myeloid leukemia and myelodysplastic syndromes in patients with multiple myeloma. Eur. J. Haematol..

[16] Gramegna D., Bertoli D., Cattaneo C., Almici C., Re A., Beloti A., Borlenghi E., Lanzi G., Archetti S., Verardi R. (2022). The role of clonal hematopoiesis as driver of therapy-related myeloid neoplasms after autologous stem cell transplantation. Ann. Hematol..

[17] Franz G., McClune B., Buadi F., Walsh W., White F., Przepiorka D. (2006). Myelodysplasia after Autologous Stem Cell Transplantation for Multiple Myeloma. Blood.

[18] Mouhieddine T.H., Sperling A.S., Redd R., Park J., Leventhal M., Gibson C.J., Manier S., Nassar A.H., Capelletti M., Huynh D. (2020). Clonal hematopoiesis is associated with adverse outcomes in multiple myeloma patients undergoing transplant. Nat. Commun..

[19] Siegel R.L., Miller K.D., Fuchs H.E., Jemal A. (2022). Cancer statistics, 2022. CA Cancer J. Clin..

[20] Díaz-Tejedor A., Lorenzo-Mohamed M., Puig N., Garcia-Sanz Ramon Mateos M.V., Garayoa M., Paino T. (2021). Immune system alterations in multiple myeloma: Molecular mechanisms and therapeutic strategies to reverse immunosuppression. Cancers.

[21] Swan D., Lynch K., Gurney M., O’Dwyer M. (2019). Current and emerging immunotherapeutic approaches to the treatment of multiple myeloma. Ther. Adv. Hematol..

[22] Smith A., Howell D., Patmore R., Jack A., Roman E. (2011). Incidence of haematological malignancy by sub-type: A report from the Haematological Malignancy Research Network. Br. J. Cancer.

[23] Wang J., Lv C., Zhou M., Xu J.Y., Chen B., Wan Y. (2022). Second Primary Malignancy Risk in Multiple Myeloma from 1975 to 2018. Cancers.

[24] Ragon B.K., Shah M.V., D’Souza A., Estrada-Merly N., Gowda L., George G., de Lima M., Hashmi S., Kharfan-Dabaja M.A., Majhail N.S. (2023). Impact of Second Primary Malignancy Post-Autologous Transplantation on Outcomes of Multiple Myeloma: A CIBMTR Analysis. Blood Adv..

[25] Palumbo A., Hajek R., Delforge M., Kropff M., Petrucci M.T., Catalano J., Gisslinger H., Jędrzejczak W.W., Zoledava M., Weisel K. (2012). Continuous lenalidomide treatment for newly diagnosed multiple myeloma. N. Engl. J. Med..

[26] Gupta S., Agrawal A., Agrawal S., Su H., Gollapudi S. (2006). A paradox of immunodeficiency and inflammation in human aging: Lessons learned from apoptosis. Immun. Ageing.

[27] Zhu B., Qu S. (2022). The relationship between diabetes mellitus and cancers and its underlying mechanisms. Front. Endocrinol..

[28] Sperling A.S., Guerra V.A., Kennedy J.A., Yan Y., Hsu J.I., Wang F., Nguyen A.T., Miller P.G., McConkey M.E., Quevedo Barrios V.A. (2022). Lenalidomide promotes the development of TP53-mutated therapy-related myeloid neoplasms. Blood.

[29] Jones J.R., Cairns D., Menzies T., Pawlyn C., Davis F.E., Sigsworth F., Jenner M.W., Kaiser M.F., Mottram M., Drayson M.T. (2022). Second Primary Malignancy Incidence in Patients Receiving Lenalidomide at Induction and Maintenance; Long-Term Follow up of 4358 Patients Enrolled to the Myeloma XI Trial. Blood.

[30] Cooper J.D., Thornton J.A., Gibson S.J., Pham K., Sunderland K., deStefano C.B. (2022). Survival of Patients with Multiple Myeloma Diagnosed with Second Primary Malignancies: An ASCO Cancerlinq Analysis. Blood.

[31] Usmani S.Z., Sexton R., Hoering A., Heuck C.J., Nair B., Waheed S., Al Sayed J., Chauhan N., Ahmed N., Atrash S. (2012). Second malignancies in total therapy 2 and 3 for newly diagnosed multiple myeloma: Influence of thalidomide and lenalidomide during maintenance. Blood.

