# Supplementary material for: Outcome of Second Primary Malignancies Developing in Multiple Myeloma Patients
Source: Cancers (Basel). 2023 Sep 1;15(17):4359. doi: 10.3390/cancers15174359 (PMC10487060; doi:10.3390/cancers15174359)
Supplement: Supplementary file 1 [file cancers-15-04359-s001.zip › cancers-2473040-supplementary.pdf]

Table S1. (A) .SPM subtypes;.

SPM subtype	Number of patientss
**Solid-SPMs**<br>Stage 1(n=-31), stage 2(n=14), stage 3-4 (n=47)	113
Bladder Cancer	9
Prostate Cancer	10
Thyroid Cancer	2
Uterine Cancer	7
Gastric Cancer Colon Cancer	8
Rare solid	6
Breast Cancer	9
Hepatoma	3
Ovarian	1
Colorectal Cancer	17
Renal Cancer	3
Lung Cancer	16
Melanoma	9
Oral and Oropharyngeal Cancer	4
Pancreatic Cancer	9
**Hematological**	52
MDS+AML+MPN	36
Lymhoblastic Leukemia*	5
Lymphoma	11
**TOTAL**	**165**

SPM – second primary neoplasm; MDS-myelodysplastic syndrome; AML-acute myeloblastic leukemia; MPN- myeloproliferative neoplasm. *including one patient with biphenotypic leukemia.

Table S1. (B) Cytogenetic and molecular changes detected in 23 patients diagnosed with AML (n=), ALL(2), MDS() and MPN(2).

Diagnosis	Genetic abnormalities
CMML	BCR/ABL Negative-; Karyotype 46,XY [5]
MDS	complex karyotupe add (3q25); del 9q (21q22); del 16q21q22)
B-ALL	tetrasomy 9,11 ,12
MDS	del7
MDS	TP53 mutation, mnosomy 5 and 12, loss of Y-Chromosome in all examed cell-metaphases
AML preceded by MDS	DNMT3A mutation, SF3B1 mutation, complex aberrant karyotype
MDS	DNMT3A mutation, SF3B1 mutation, EZHZD mutation, TET2 mutation, complex aberrant karyotype
B-ALL	Ph negativ, Del9p21 (CDKN2A-gene), Del20q12 (PTPRT), Deletion long arm of Chromosome 9, Monosomy 20, and Trisomy 21
AML preceded by MDS	RUNX1 mutation, EZH2 mutation, complex aberrant karyotype
AML preceded by MDS	Addition of 2 copies in region 11q22.3 (ATM) no other aberrations were found.
Biphenotypic leukemia preceded by MDS	RUNX1 mutation, EZH2 mutation, complex aberrant karyotype.
MDS	karyotype: 43,XX,del(5q),-7,der(17)del(17)(p13)t(13;17)(q14;p13),der(21q;18q)[8]/46,XX[38]. nuc ish(TP53x1,MPOx2)[43/85]// Molecular finding: Frecuencia alélica 8,4 % Gen TP53. c.578A>G(NM_000546.5)(p.H193R)
MDS	karyotype: 45,XX,-7,t(9;22)(q34;q11)[3]/45,X,t(X;11)(q13;p15),-7,t(9;22)(q34;q11)[19]/45,X,del(X)(q?),-7,t(9;22)(q34;q11)[5]/ 46,XX,-7,t(9;22)(q34;q11)+mar[3]//molecular finding: Ratio BCR-ABL1/ABL1 p210 (IS): 12.4931, Gen WT1: cuantificación de expresión (%): 220.37

MDS	karyotype: 46,XY,der(3)t(3;6),del(5q),-6,del(6q),der(7)t(3;7)del(7q13q36),add(16p),add(17p),der(21)[cp14]/46,XY[11]// molecular finding: Frecuencia alélica 24.75%. Gen TP53. c.733G>A(NM_000546.5)(p.G245S)
MDS	46,XY, del (7)(q11,2q22)[3]
AML preceded by MDS	45 ~ ?47 ,XX,-5,del(6)(q2? lq2?5),-7 ,add(8)(q24), +marł, +mar,inc[cp9] .
AML preceded by MDS	46,XY[3].nuc ish(TP53,D17Zl)x2[500]
AML preceded by MDS	43 ,X,-Y ,add(7)(p22),-13 ,-16,add(l 7)(p 11.2) ,-19 ,-21, +marł,+ mar2, + mar3, +mar4, +mar5, + mar6,+ 2 ~ 3mar[cp 12]/43,X,-Y ,add(7)(p22),add(13)(p 11.2),-16,-17 ,add(l 7)(pl 1.2),-19,-21, +marł, +mar2, +mar3,+ mar4, + 1 ~ 4mar[cp10]/43,X,-Y ,del(2)(q3? 1),add(7)(p22),add(l l)(p 15),der(13; 13)(q 10;q10),-16,add(l 7)(pl 1.2), add(l 7)(pl l .2),-19,-2 l, +marł, +mar2, +mar4[cp3]/43,X,-Y,add(7)(p22),add(12)(pl3),-13,-16,-17,add(l 7)(pl 1.2),-19,-21, +marł, +mar4, +mar5[2]/46,XY[5].
AML	46,XY
ET	No BCR-ABL1 fusion transcript; c.1849G>T (p.V617F) mutation in JAK2 gen in 98% of allelles in the sample
MDS	karyotype:<br>45,XX,-3,del(5)(q14q34),der(14)t(14;17)(p13;q11),-17,+r[2]<br>46,sl,+21[4]<br>44,XX,-3,del(5),-7,der(12;14)(q10;q10),+r[4]<br>46,XX[1]
AML	karyotype:<br>45,XY,-7,del(12)(p11p12),del(13)(q13q21)[6]<br>46,XY[4]
MDS	karyotype:<br>43-46,XY,der(3;7)(q10;q10)[2],-5[5],-7[1],add(7)(p11)[3],-17[5],-19[3],del(21)(q11q21),+r[1],+mar1[4],+mar2[1][cp6]<br>46,XY[4]

Cytogenetic/ molecular data were available for 23 out of the 41x patients diagnosed with these malignancies. B-ALL- B acute lymphoblastic leukemia; AML- acute myeloid leukemia; CMML chronic myelomonocytic leukemia;-;ET-essential thrombocytosis; MDS - myelodysplastic syndrome; MPN - myeloproliferative neoplasms.

Table S2. (A). Univariate analysis - Factors Predicting time to Solid-SPM since MM diagnosis.

	HR	CI95%	P
Age <65 ≥65 (years)	2.77	1.84-4.15	**<0.001**
Sex (Females)			
Males	1.45	0.99-2.15	0.053
Prior cancer history	2.03	1.05-3.93	**0.034**
Concomitant T2DM	1.65	1.05-2.60	**0.029**
Number of concomitant comorbidities (0)			
1	1.63	1.00-2.67	0.05
2	1.48	0.88-2.49	0.14
3	1.31	0.73-2.34	0.37
Creatinine level ≥ 1.3(mg/dL)	1.10	0.71-1.68	0.68
Hemoglobin >=12 (gr/dL)	0.72	0.47-1.08	0.11
Platelets >150 (10^9/L)	0.59	0.34-1.04	0.067
Albumin level >=3.5 (g/dL)	0.74	0.49-1.14	0.17
B_2Mmicroglobulin >= 5.5 (mg/L)	1.81	1.14-2.87	**0.012**
ISS (1)			
2	1.27	0.75-2.14	0.37
3	1.63	0.97-2.74	0.067
Any prior chemotherapy for MM	0.68	0.45-1.02	0.06
Any prior IMiD	0.86	0.50-1.50	0.6
Any prior PI	1.02	0.66-1.57	0.93
Any maintenance therapy	1.11	0.75-1.64	0.61
Maintenance with IMiD	0.97	0.64-1.48	0.89
Prior AutoHCT	0.65	0.44-0.96	**0.03**
Number of prior lines ≤2 vs ≥3	0.38	0.25-0.58	**<0.001**

ECOG PS (0)			
1	1.47	0.87-2.47	0.15
2	2.01	1.09-3.69	**0.025**
3	0.69	0.3-1.58	0.38
4	0.47	0.06-3.53	0.47

AutoHCT- Autologous hematopoietic cell transplantation; B_2M- B_2 Microglobulin; ECOG PS - ECOG Performance Status; IMiD- immunomodulating agents; ISS- International Staging System; MM- multiple myeloma; PI- proteasome inhibitor; SPM-second primary malignancy;T2DM-. Type 2 diabetes mellitus. Hemoglobin, platelets, albumin, creatinine, B_2 Microglobulin levels were measured prior to the initiation of any anti-MM treatment.

Table S2. (B) Univariate analysis - Factors Predicting Shorter time to HematoSPM only.

	HR	CI95%	P
Age <65			
⩾65(years)	2.14	1.21-3.80	**0.009**
Sex (F)			
M	0.66	0.36-1.24	0.20
Prior cancer history	3.39	1.18-9.71	**0.023**
Concomitant T2DM	2.63	0.99-6.98	0.052
Number of concomitant comorbidities			
0	0.41	0.21-0.81	
1	1.82	0.87-3.78	0.0.1
2	3.61	1.57-8.30	**0.003**
3	4.89	1.74-13.7	**0.003**
Hemoglobin >=12 (gr/dL)	0.66	0.32-1.39	0.28
Creatinine level ⩾ 1.3 (mg/dL)	1.00	0.48-2.07	>0.99
PLT >150 (10^9/L)	0.37	0.16-0.86	**0.021**
Albumin level >=3.5 (g/dL)	1.27	0.63-2.56	0.51
B2Mmicroglobulin >= 5.5 (mg/L)	0.96	0.46-1.99	0.91
ISS 1			
2	1.20	0.51-2.82	0.67
3	1.09	0.5-2.37	0.83
Any prior anti-MM chemotherapy	0.91	0.46-1.81	0.79
Any prior IMiD	1.06	0.47-2.36	0.89
Any prior PI	1.28	0.59-2.75	0.53
Any maintenance therapy	1.17	0.67-2.06	0.57
Maintenance with IMiD	0.51	0.28-0.95	**0.032**
Prior AutoHCT	1.05	0.59-1.88	0.87
Number of prior lines 1,2 vs >2	0.43	0.24-0.77	**0.005**
ECOG PS 0 VS ⩾ 1	0.45	0.2-1.00	0.05

AutoHCT- Autologous hematopoietic cell transplantation; B2M- B2 microglobulin; Chemo-chemotherapy; ECOG PS - ECOG Performance Status; IMiD- immunomodulating agents; ISS- International Staging System; PI- proteasome inhibitor; PLT- platelets; SPM-second primary malignancy; T2DM-type 2 diabetes mellitus. Hemoglobin, platelets, creatinine, albumin and B2M levels were measured treatment prior -initiation of any anti- MM treatment. ECOG PS was determined at SPM diagnosis.

Table S3. Management of SPMs.

	**Number of Patients (%)**
**Solid SPMs**	**113**
Supportive	25
Any antineoplastic therapy	88
Surgery only	37
Surgery+ Radiotherapy	10
Surgery+ Systemic therapy	
Chemotherapy	11

Biological	1
Radio only	7
Systemic only	
Chemotherapy	13
Biological	7
Systemic+ Radiotherapy	2
**Hematological SPMs (52)**	**52**
Supportive	7
Watch and Wait	7
Any systemic antineoplastic therapy	38
Chemotherapy	31
Biological	3
Chemotherapy+ Radiotherapy	2
Chemotherapy followed by AlloHCT	2

AlloHCT- Allogeneic hematopoietic cell transplantation; SPM –Second primary malignancy.

Table S4. (A) Detailed characteristic of MM management in response to SPM detection.

MM Treatment at the time of SPM diagnosis	MM treatment Post SPM	Entire cohort[&] (n=100)	Solid cohort[&] (n=75)	Hemato cohort[&] (n-=25)
**IMiD** (n=55);solid-40; Hem-15	Discont	33	20	13
	Substituted	3	1	2
	Cont	19	19	0
**IMiD-Chemo** (n=5);solid-5;Hem-0	Discont	3	3	0
	Substituted	0	0	0
	Cont	2	2	0
**PI** (n=7); solid-5; Hem-2	Discont	6	5	1
	Substituted	0	0	0
	Cont	1	0	1
**IMiD-PI** (n=5); solid-4; Hem-1	Discont	2	1	1
	Substituted	0	0	0
	Cont	3	3	0
**MoAB** (n=8); solid-6; Hem-2	Discont	5	3	2
	Substituted	0	0	0
	Cont	3	3	0
**Chemotherapy** (n=4); solid-2, Hem-2	Discont	3	1	2
	Substituted	0	0	0
	Cont	1	1	0
**Other**	Discont	0	1	0
	Substituted	0	0	0
	Cont	1	0	0
**Not Determined** (n=15); solid-12, Hem-3	Discont	0	0	0
	Substituted	12	9	3
	Cont	3	3	0

MM – multiple myeloma; SPM – second primary malignancy; IMiD – immunomodulatory drugs, PI – proteasome inhibitors, MoAB – monoclonal antibody. 65 were not receiving anti-MM therapy at the d time of SPM diagnosis, including 38 /113 in the solid cohort and 27/52 in the Hemato cohort.

Table S4. (B) . Univariate analysis - Factors Predicting OS since SPM diagnosis for the entire cohort (n=165).

	HR	CI95%	P
Age <65			
≥65	1.09	0.72-1.64	0.68
Sex F			
M	0.74	0.49-1.12	0.16
Prior cancer history	1.70	0.85-3.40	0.13
Any prior Chemo	1.29	0.80-2.08	0.30
Any prior IMiD	1.45	0.78-2.69	0.24
Any prior Pi	1.02	0.65-1.60	0.95
Any maintenance therapy	0.57	0.37-0.90	**0.015**
Prior AutoHCT	0.68	0.44-1.03	0.067
Number of prior lines 1,2 vs >2	1.61	1.07-2.43	**0.022**
Creatinine level <1.3	0.59	0.37-0.93	**0.023**
≥ 1.3 (mg/dl)			
PLT <150			
>150 (G/L)	0.74	0.40-1.35	0.32
SPM type Solid			
Hemato	1.25	0.80-1.96	0.33
Albumin level <3.5			
>=3.5 (g/dl)	0.83	0.52-1.32	0.43
B2Mmicroglobulin <5.5	0.63	0.38-1.04	0.069
>= 5.5			
ISS 1			
2	1.43	0.78-2.63	0.25
3	1.71	0.97-3.00	0.064
ECOG PS 0			
1	3.00	1.42-6.36	**0.004**
2	5.86	2.68-12.8	**<0.001**
3	6.80	2.59-17.8	**<0.001**
No concomitant comorbidities	0.75	0.47-1.19	0.23
Concomitant comorbidities 0			
1	1.70	1.0-2.89	0.05
2	1.12	0.62-2.0	0.71
3	1.12	0.60-2.11	0.72
Concomitant diabetes	1.29	0.78-2.12	0.32
Concomitant HT	0.96	0.64-1.45	0.85
Hemoglobin <12			
>=12 (g/dl)	0.52	0.32-0.86	**0.010**
Maintenance after 1 Line	0.58	0.35-0.96	**0.036**

Table S4. (C). Univariate analysis - Factors Predicting OS since SPM diagnosis (excluding MM deaths).

	HR	CI95%	P
Age <65			
≥65 (years)	1.11	0.7-1.76	0.65
Sex (F)			
M	0.77	0.48-1.22	0.26

Prior cancer history	1.95	0.84-4.52	0.12
Any prior Chemotherapy	1.24	0.72-2.15	0.43
Any prior IMiD	1.58	0.77-3.24	0.21
Any prior PI	0.85	0.51-1.43	0.54
Any maintenance therapy	0.60	0.37-0.98	**0.041**
Prior AutoHCT	0.64	0.40-1.02	0.062
Number of prior lines 1,2 vs >2	1.41	0.88-2.25	0.15
Creatinine level ≥ 1.3 mg/dL	1.73	1.05-2.85	**0.032**
Platelets >150 10^9/L	0.82	0.43-1.59	0.57
SPM type (solid)			
Hematological	1.13	0.68-1.88	0.63
Albumin level			
>=3.5 (g/dL)	0.86	0.51-1.46	0.58
B_2Mmicroglobulin			
>= 5.5 (mg/L)	1.54	0.89-2.66	0.12
ISS (1)			
2	1.37	0.68-2.78	0.38
3	1.74	0.91-3.33	0.10
ECOG PS 0 vs ≥1	0.26	0.12-0.55	**<0.001**
	2.91	1.30-6.52	**0.009**
	5.42	2.35-12.5	**<0.001**
	7.88	2.8-22.2	**<0.001**
Concomitant comorbidities 0	0.75	0.44-1.29	0.3
1	1.73	0.94-3.16	0.077
2	1.05	0.54-2.07	0.88
3	1.17	0.58-2.36	0.66
Concomitant T2DM	1.23	0.71-2.13	0.45
Hemoglobin >=12 g/dL	0.51	0.30-0.89	**0.017**
Any maintenance therapy	0.54	0.32-0.092	**0.023**

AutoHCT- Autologous hematopoietic cell transplantation; B2M- B_2 Microglobulin; ECOG PS - ECOG performance status; HT- hypertension ;IMiD- immunomodulating agents; ISS- International Staging System; T2DM- Type 2 diabetes mellitus; PI- proteasome inhibitor; SPM-second primary malignancy.
